# Mutual antagonism between hepatitis B viral mRNA and host microRNA let-7

**DOI:** 10.1038/srep23237

**Published:** 2016-03-16

**Authors:** Akemi Takata, Motoyuki Otsuka, Motoko Ohno, Takahiro Kishikawa, Takeshi Yoshikawa, Kazuhiko Koike

**Affiliations:** 1 Department of Gastroenterology, Graduate School of Medicine, The University of Tokyo, Tokyo 113-8655, Japan; 2 Japan Science and Technology Agency, PRESTO, Kawaguchi, Saitama 332-0012, Japan

## Abstract

The interplay between viral and host factors plays a major role in viral pathogenesis. Hepatitis B virus (HBV) infection is a global health problem that leads to liver cirrhosis and hepatocellular carcinoma (HCC). Although HBV proteins have been studied extensively about their implication in hepatocarcinogenesis, the molecular mechanisms of oncogenesis are still largely unknown. A recent concept in gene regulation, in which competitive endogenous RNAs compete for common microRNAs (miRNAs), suggests that mRNA targets are key elements in the regulation of miRNA availability. Here, we show that HBV mRNA in the preS2 region can be targeted by host miRNA let-7 g. This leads to the sequestration of let-7 g and inhibition of let-7 g function. The expression of HBV transcripts, including the preS2 region, de-repressed let-7 g targets, which may contribute to long-term oncogenesis. HBV transcript-expressing transgenic mice, but not non-targeted transcript-expressing mice, were more prone to chemically induced hepatoocarcinogenesis. Let-7 target protein expression was upregulated in human HCC tissues derived from HBV-infected patients. On the other hand, let-7 g inhibited HBV preS2 protein expression and viral products. These results suggest that the interplay between viral intermediate transcripts during HBV replication and host miRNAs is crucial to the pathogenesis of chronic viral infection.

MicroRNAs (miRNAs) are short, single-stranded, non-coding RNAs. Mature miRNAs are recruited into the Ago2-related RNA-induced silencing complex (RISC) and act as suppressors of gene expression. Although they cannot be used for completely accurate prediction of target sequences, “seed” sequences consisting of 2–7 nucleotides within miRNAs are considered critical for selecting targets^1^. Depending on the target mRNA, miRNAs are responsible for various biological functions, including oncogenesis and oncogenic suppression^2^,^3^,^4^.

Although pseudogenes had been recognized as defunct relatives of known genes, some pseudogenes, such as the phosphatase and tensin homology deleted on chromosome ten (PTEN) pseudogenes, have been recently reported as biologically functional. These decoys consume copies of the miRNAs, de-repressing PTEN and enhancing its tumor suppressor activity^5^. These decoys consume copies of the miRNAs, de-repressing PTEN and enhancing its tumor suppressor activity^5^. Similar competitive endogenous RNAs have been reported, and these networks may play important trans-regulatory functions^6^.

More than 350 million people globally are chronic carriers of the Hepatitis B virus (HBV)^7^. A significant number of these carriers suffer from either liver failure or hepatocellular carcinoma (HCC) during the late stages of the disease^8^. HBV is a DNA virus with a 3.2-kb-long partially double-stranded relaxed circular DNA (rcDNA) as its genome that contains four open reading frames for the P (DNA polymerase/reverse transcriptase), C (core protein), S (surface protein), and X (X protein) genes. The S gene is divided into preS1 and preS2 regions and the small S gene, and the HBV envelope is composed of three HBV surface antigen (HBsAg) forms: large S (coded for by the pre-S1/pre-S2/S gene), middle S (the preS2/S gene), and small S (the S gene) proteins. Because HBV sequences have diversities, genotypes of HBV have been recognized by a sequence divergence of more than 8% in the entire genome and named by capital alphabetical letters in the order of discovery^9^ with distinct geographic distribution^10^. HBV genotype A is prevalent in Africa, Europe and India, genotype B and C are common in Asia, and genotype D is distributed all over the world^10^.

HBV replication process includes the step of reverse transcription. Covalently closed circular HBV DNA (cccDNA) is formed by conversion from rcDNA after HBV infection and exists persistently in the hepatocyte nucleus in an episomal state, where it acts as a viral transcription template. Transcribed RNAs, which serve as mRNAs for the viral proteins or serve as a template for the viral genome DNA through reverse transcription using viral reverse transcriptase. Nucleocapsids containing rcDNA are released from the host cell as virions or are converted to cccDNA in the nucleus. While nucleos(t)ide analogs efficiently suppress HBV replication^11^, complete elimination of cccDNA remains difficult^12^.

In general, viral and cellular miRNAs involved in host-pathogen interactions are engaged during the host-viral offense and defense^13^,^14^,^15^,^16^. Through these interactions, viruses may establish an environment favorable for their persistence, which may be pathogenic to the host, and, at the same time, host may work as defending such viral infection. In this study, we hypothesized that, besides HBV mRNAs function as templates for the viral products, they may work like cellular pseudogenes that function as decoys of cellular miRNAs^17^,^18^,^19^, leading to deregulate the cellular circumstances and provide with one of the pathogenic molecular mechanisms. In addition, we determined whether host cellular miRNAs possibly interacting with the viral RNA may interfere the HBV replication.

## Results

### HBV preS2 mRNA interacts with cellular microRNA let-7 g

**Figure 1:**
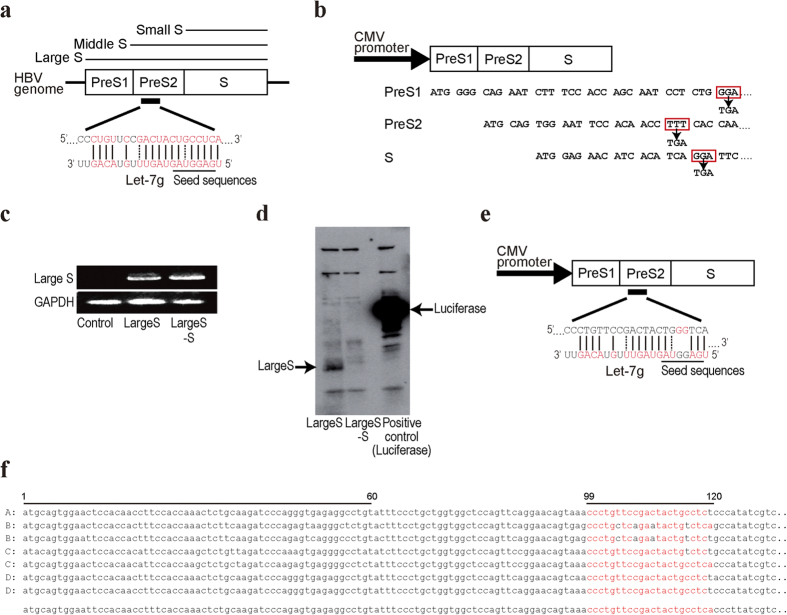
HBV preS2 mRNA can be targeted by let-7. (**a**) PreS2 mRNA is targeted by let-7 g. The possible target preS2 mRNA and let-7 g sequences are shown. Complementary sequences are indicated in red. Dashed lines indicate wobble complementarity. Positions of the let-7 g seed sequences are also indicated. Three forms of HBV surface antigen (HBsAg): large S (coded for by the pre-S1/pre-S2/S gene), middle S (the preS2/S gene), and small S (the S gene) proteins are also indicated. (**b**) CMV promoter-driven Large S expressing construct (“Large S” construct) is shown. To prevent protein translation, three stop codons were introduced by mutagenesis into the preS1, preS2, and S regions, indicated in red rectangles (“Large S–S” construct). The codons were changed to TGA, a stop codon. (**c**) Expression of Large S and Large S–S transcripts was confirmed. RNAs at 48 h after transfection into Huh7 cells with the indicated constructs were extracted and subjected to RT-PCR. (**d**) *In vitro* translation confirmed no protein translation from the Large S–S construct. Luciferase protein was used as an experimental control. (**e**) Large S–S construct with mutations in the let-7 g seed sequences is shown. Two bases corresponding to the let-7 g seed sequences in the Large S transcript-expressing construct were mutated (Large S-SM). (**f**) Representative HBV preS2 sequences derived from genotypes A–D are shown. The genotype D HBV DNA sequences from HepG2.2.15 cells used in this study are shown at the bottom. Potential let-7 g-targeting sequences, shown in red, are from nucleotides 99 to 120, and the nucleotides differing from the sequences used in this study are shown in black. Frequently deleted regions, which are in preS2 from nucleotides 1 to 60, are also indicated. The GenBank accession numbers for the HBV sequences shown here are as follows: genotype A, AB116093; genotype B, AB073853 and AB073828; genotype C, AB105174 and X52939; and genotype D, X80926 and AB205126.

To determine whether HBV mRNA can functionally interact with host miRNAs, HBV genotype D mRNA sequences were examined *in silico* for possible miRNA-targeting sequences using the miRanda database. While several sequences were selected as candidates, sequences in the HBV preS2 region of the HBV large S antigen had the highest probability according to another confirmatory search using the RNA22 database (Supplementary Figure 1). These sequences are targeted by let-7 g, with complementarity at positions 1–13 from the miRNA 5′-end, including the seed region, and 83% complementarity of the entire miRNA sequence (Fig. 1a).

To examine the biological function due to these interactions further, we cloned a plasmid encoding the HBV large surface antigen (Large S) using genotype D HBV genomic sequences derived from HBV-integrated HepG2.2.15 cells^20^. To exclude the effects of HBV proteins, we constructed a plasmid expressing Large S mRNA that cannot synthesize the protein by introducing stop codons (named “Large S–S”) via mutagenesis at the earliest positions from the start codons of the preS1, preS2 and S genes each (Fig. 1b). The transcripts from the Large S and Large S–S constructs were confirmed by reverse transcription-polymerase chain reaction (RT-PCR) (Fig. 1c). Protein expression from the Large S construct and no protein expression from the Large S–S construct were both confirmed by *in vitro* transcription and translation (Fig. 1d). In addition, a construct with mutations in the preS2 sequences that disrupts the complementarity to the seed sequences of let-7 g was generated from the Large S–S construct (named as “Large S-SM”) (Fig. 1e). Because HBV sequences show diversity across the genotypes, we extracted and aligned representative HBV sequences from the Hepatitis Virus Database^21^. We confirmed that the corresponding sequences are mostly conserved irrespective of the genotypes (Fig. 1f). In addition, while frequent deletion of the preS region was reported, particularly in cases with hepatocellular carcinoma (HCC)^22^,^23^,^24^, we confirmed that such deletions usually occur at the terminal of preS1 and starting regions of preS2, which do not overlap with the let-7 g targeting sequences (Fig. 1f).

### HBV large S mRNA suppress let-7 g function

**Figure 2:**
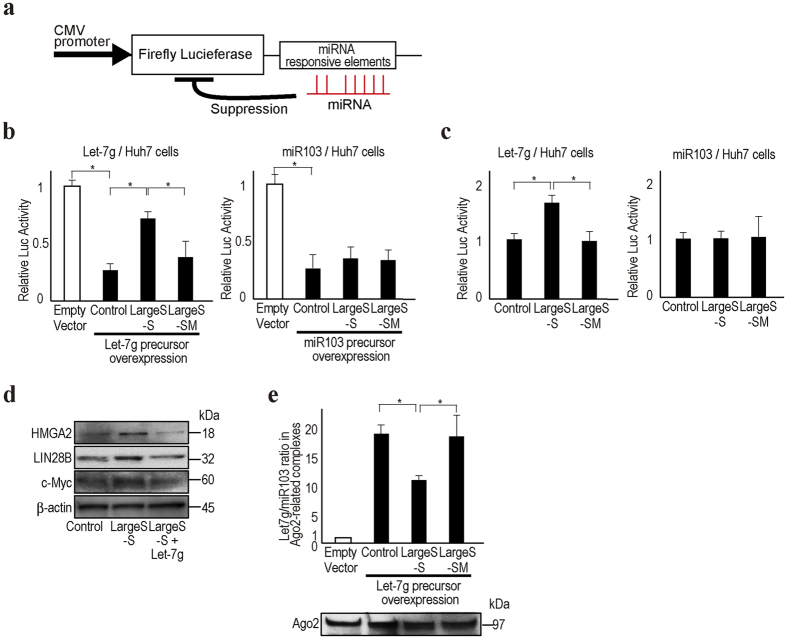
HBV preS2 mRNA inhibits let-7 g function. (**a**) The CMV promoter-driven firefly reporter construct is shown. The reporter contains miRNA-responsive elements in its 3′UTR. Luciferase expression is suppressed by the miRNAs. (**b**) Huh7 cells were transiently transfected with the indicated miRNA reporter constructs (pGL4-let-7 g or pGL4-miR103) with or without corresponding miRNA precursor-overexpressing plasmids (pCDH-let-7 g or pCDH-miR103). Luciferase activity was significantly suppressed when overexpressing miRNA precursors (“Control” compared to “Empty vector” which means without miRNA overexpressing plasmid). Expression of Large S–S (pCDH-Large S–S) reversed such suppression by inhibiting let-7 g function (left), but not in case of miR103 (right). The Large S-SM construct (pCDH-Large S-SM) did not show such effects. Data represent the means ± s.d. of three independent experiments, and the values from the cells without miRNA overexpression were set as 1. *p < 0.05. (**c**) Similar to the description in b, Large S–S inhibited endogenous let-7 g function (left), but not miR103 function (right). In this case, to examine the effects of LargeS-S or Large S-SM construct on the function of endogenous miRNAs, miRNA overexpressing plasmids were not used. Data represent the means ± s.d. of three independent experiments, and the values from the control were set as 1. *p < 0.05. (**d**) The expression levels of let-7 g target proteins were upregulated in Large S–S stably expressing Huh7 cells according to Western blotting. Forced stable expression of let-7 g in Large S–S-expressing Huh7 cells canceled the effects of Large S–S expression. Representative results from three independent experiments are shown. (**e**) Let-7 g loading into the Ago2-associated RISC was decreased by Large S–S expression but not by Large S-SM expression. miRNA levels were quantitated in the RISC after immunoprecipitation of Ago2. Equal precipitation of Ago2 was confirmed by Western blotting (bottom). Data are shown after normalizing the let-7 g levels to miR103 levels in the Ago2-associated complexes. The values of the sample without let-7 g overexpression were set as 1. Data represent the means ± s.d. of three independent experiments. *p < 0.05.

To determine the functional changes in miRNAs in cells with HBV transcripts harboring the preS2 region, we used luciferase-based reporters containing miRNA responsive elements (Fig. 2a). With overexpression of miRNA-expressing plasmids corresponding to the reporters in Huh7 cells, the luciferase values were significantly reduced by the expressed miRNA function (“Control” in Fig. 2b). However, when using let-7 g reporter and precursor constructs, simultaneous expression of the Large S–S construct significantly suppressed the let-7 g function, and luciferase values were recovered (Fig. 2b). These effects were not observed when expressing the Large S-SM construct, which has mutations in the complementary regions of the let-7 g seed sequences, suggesting that the effects were let-7 g-specific. The suppression of miRNA function by the Large S–S construct was not observed when using the miR103 reporter or precursor constructs (Fig. 2b), again suggesting specificity to let-7 g function. These phenomena were similarly observed even without exogenous miRNA precursor expression (Fig. 2c) and in HepG2 cells (Supplementary Figure 2). As expected, the protein expression levels of HMGA2, LIN28B, and c-myc, which are let-7 g targets, were increased in Large S–S-expressing cells (Fig. 2d).

Because the results suggest that let-7 g may be sequestered by HBV large S transcripts, we examined the let-7 g levels in Ago2-associated RISC using RNA immunoprecipitation (RIP), adjusted for the miR103 levels in RISC, which were measured simultaneously as a control. To visualize the results more easily by enhancing the basal effects, let-7 g precursor-expressing cells were used for this assay. Although the precipitated Ago2 protein levels were almost unchanged, let-7 g levels in RISC were approximately 20-fold higher than those in the control cells stably transfected with a control vector (Fig. 2e). However, the let-7 g levels in RISC were reduced by ~50% when using the cells stably expressing the let-7 g precursor construct and Large S–S. These effects were not observed when using the cells stably expressing the let-7 g precursor construct and Large S-SM, suggesting that the Large S transcripts sequestered let-7 g from RISC through their interactions and reduced let-7 g intrinsic function.

### Large S transcript transgenic mice are prone to chemically-induced liver tumor

**Figure 3:**
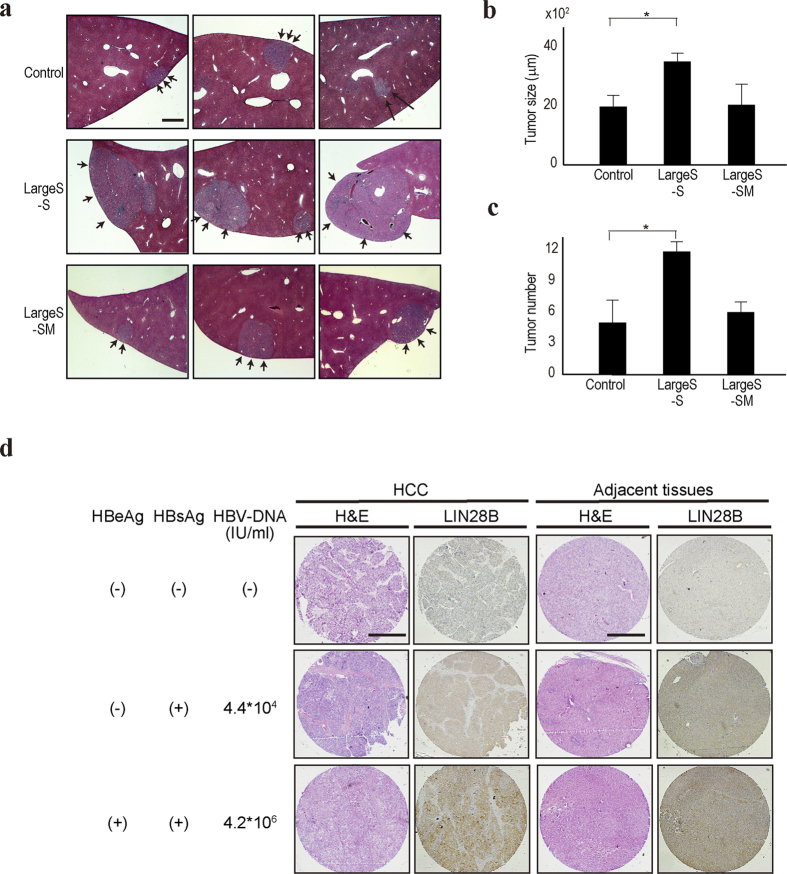
Large S–S transgenic mice were prone to DEN-induced hepatocarcinogenesis. (**a**) Representative H&E-stained liver tissues from control, Large S–S transgenic, and Large S-SM transgenic mice are shown. The mice were sacrificed 16 weeks after DEN treatment. Arrows indicate the tumors in the liver. Bar, 500 μm. (**b**,**c**) The number and size of the liver tumors from each mouse group are shown. Six mice were used in each group. Data represent the means ± s.d. of three independent experiments. *p < 0.05. (**d**) LIN28B protein expression levels in the HCC tissues and their surrounding tissues derived from HBV-infected or non-infected patients were determined by immunohistochemistry. Representative images including H&E staining, along with the patient clinical information (the positivity of HBeAg and HBsAg, and the HBV-DNA load in the sera), are shown. The stained proteins are in brown. Bar, 500 μm.

To examine the biological effects of Large S transcripts *in vivo*, we constructed transgenic mice expressing HBV Large S–S transcripts and Large S-SM. Although no spontaneous liver tumors were observed in these mice, mice transfected with the HBV Large S–S construct had more and larger liver tumors after treatment with diethylnitrosamine (DEN) to enhance tumorigenesis (Fig. 3a–c, and Supplementary Figure 3a). However, the results from mice expressing the Large S-SM construct were comparable to those from control mice (Fig. 3a–c), suggesting that the sequences in Large S are crucial for these phenotypes. Additionally, to examine the expression levels of LIN28B, a let-7 target gene, in liver tissues derived from patients with HBV infection, immunohistochemistry was performed using HCC and the surrounding tissues from HBV-infected and -uninfected cases. The LIN28B protein expression levels were significantly higher in HCC tissues and their surrounding tissues derived from the patients with HBV infection than in those from non-infected patients (Fig. 3d and Supplementary Figure 3b,c). Particularly, samples from patients with a high HBV load express higher levels of LIN28B protein in HCC adjacent liver tissues. These results support the *in vitro* results that HBV transcripts may suppress let-7 function and, in those cases, let-7 target protein expression is upregulated.

### Let-7 g antagonizes HBV replication

**Figure 4:**
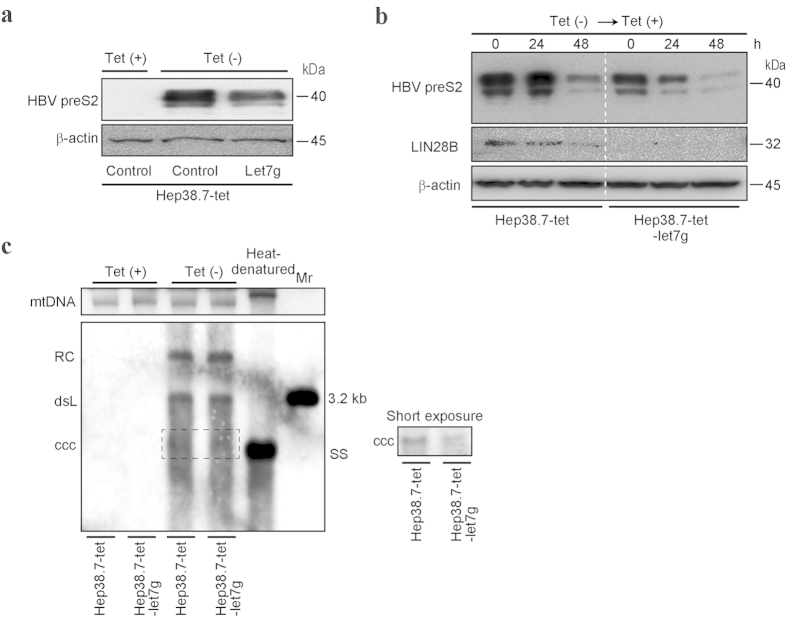
Let-7 g decreases HBV preS2 protein expression. (**a**) Hep38.7-tet cells were cultured long-term with or without tetracycline (Tet). HBV preS2 protein expression was determined by Western blotting. Let-7 g overexpression suppressed preS2 protein levels. Representative results from three independent experiments are shown. (**b**) Hep38.7-tet and stable let-7 g-overexpressing Hep38.7-tet cells were cultured without tetracycline (Tet), and then HBV transcription was shut off by adding tetracycline (Tet). PreS2 protein levels were determined at the indicated time points by Western blotting. LIN28B expression levels were also evaluated. Representative results from three independent experiments are shown. (**c**) HBV cccDNA levels in the indicated cells with or without Tet were determined by Southern blotting. DNA extracted by the Hirt method was applied. Mitochondrial DNA (mtDNA) was used as the loading control. Heat-denatured DNA indicated the disappearance of double-stranded DNA. Double-stranded linear full-length HBV DNA (dsL) was used as a 3.2 kb marker (Mr). RC, relaxed circular DNA. ccc, cccDNA. SS, single stranded DNA. A short-exposure image is also shown to identify cccDNA (right). Representative results from three independent experiments are shown.

To examine whether the functional interactions have any effect on HBV replication, we used the tetracycline-regulated HBV replication cell culture system (Hep38.7-tet). In these cells, tetracycline shuts off HBV expression, while the removal of tetracycline induces the expression of HBV products as well as HBV replication rapidly and efficiently^25^,^26^ (Fig. 4a). In these cells, let-7 g-overexpressing Hep38.7 cells expressed lower levels of HBV preS2 protein after long-term culture without tetracycline (Fig. 4a). In addition, when HBV products expressed from the cellular genome were suppressed by adding tetracycline after culturing the cells without tetracycline, HBV preS2 protein, although its expression was lower to begin with due to let-7 g overexpression, was decreased more rapidly in let-7 g-overexpressing Hep38.7 cells than in control Hep38.7 cells. Figure 4b, suggesting that cellular let-7 g has suppressive effects on HBV protein levels. While the role of Large S protein in cccDNA amplification is still controversial^27^,^28^, we examined the levels of cccDNA by Southern blotting with and without let-7 g overexpression in Hep38.7-tet cells. As shown in Fig. 4c, the cccDNA levels were lower in let-7 g-overexpressing cells than in control Hep38.7-tet cells in cultures without tetracycline (Fig. 4c), suggesting that let-7 g has, albeit slightly, suppressive effects on the HBV cccDNA levels.

## Discussion

In addition to the host mRNAs, exogenous RNAs, including RNAs produced from virus which infects human cells, may also be targeted by host cellular miRNAs^29^. In this study, we describe that sequences in HBV preS2 region can be targeted by cellular let-7 g, resulting in the impaired function of this miRNA through the decreased intrinsic recruitment of the miRNA into Ago2-related complexes. Simultaneously, on the part of the effects of miRNA to the virus, let-7 g overexpression decreases the HBV preS2 protein levels and possibly HBV cccDNA levels. These mutual and functional interactions between viral transcripts and host cellular non-coding RNAs may provide with novel insights in understanding the pathogenesis of chronic HBV infection.

Let-7 is a well-regarded tumor-suppressive miRNA^30^. Indeed, let-7 family members are often repressed in human cancers including HCC, promoting transformation by repressing targets such as LIN28B, HMGA2, and c-Myc, which are involved in oncogenesis^31^,^32^,^33^. We identified that the sequences in the HBV preS2 RNA can sequester let-7 g, which, in turn, impairs the intrinsic let-7 g function. In fact, the protein expression levels of LIN28B were upregulated by the existence of HBV preS2 transcript, which were antagonized by the forced expression of let-7 g. Although let-7 g is one of the twelve let-7 family members^30^, because LIN28B blocks the maturation of all let-7 family members^34^,^35^, the increased LIN28B expression may lead to repression of all miRNAs in the let-7 family, leading to a concomitant increase of let-7 targets. This feedback loop may be involved in promoting liver tumorigenesis, in which LIN28B is frequently highly expressed^36^,^37^. Therefore, suppression of intrinsic function of even only let-7 g by preS2 transcript may be one of the causative factors for long-term hepatocarcinogenesis during chronic HBV infection.

Generally, HBV infection is a high risk factor for HCC. Even during antiviral therapy, a proportion of patients develop HCC. Furthermore, some patients whose serum HBV DNA levels are under the detection limit level and ALT levels are within normal range develop HCC. Clinical studies have shown that the serum HBsAg levels are the major determinants of HCC risk, particularly in lower viremic patients^38^,^39^. In fact, none of the patients who achieved HBsAg seroclearance were found to develop HCC^40^, although HBsAg seroclearance is not common. From these data, HBsAg is apparently a causative factor for HCC occurrence, with HBs transgenic mice developing HCC^41^,^42^. However, as described in this study, preS2 transcript, not protein, may be a major causative factor for the liver tumorigenesis. Therefore, it is possible that the serum HBsAg levels are just surrogate markers as the major determinants of HCC risk and the causative factor is actually HBs mRNA levels in the hepatocytes, which cannot be measured currently without invasive liver biopsy.

The preS region is frequently deleted or mutated, particularly in HCC patients^23^,^24^,^43^,^44^. However, such deletions or mutations are mostly concentrated in the region from the C-terminus part of preS1 and to the N-terminus part of the preS2 (about 60 bp from the preS2 start site), which are outside of the sequences of preS2 focused (at 99 bp from the start site) in this study. It may be interesting to examine how much pseudogene transcripts, without protein translation due to the deletion of the preS2 start codon or nonsense mutations, exist in infected hepatocytes, and whether those viral transcripts (“viral non-coding RNAs”) are biologically functional.

Host miRNAs can inhibit, or occasionally enhance, viral replication by targeting viral RNAs^13^. From this point, let-7 g indeed inhibited preS2 protein levels in the HBV product-inducible system both stably and after shutting off the transcription of the viral products. Although the precise mechanisms of the anti-viral effects by the host microRNAs observed remain to be determined, it suggested that host microRNAs can inhibit HBV replication to a certain level in the infected cells, which may be one of the factors for maintaining the viral levels at a certain level during chronic infection. Comparing to the decreased levels of preS2 protein, the decrease of cccDNA levels was only marginal. Because the role of large S protein on the cccDNA amplification is still controversial^27^,^28^,^45^, the effects of let-7 g on cccDNA production may be complexed. The inhibitory effects on cccDNA production by let-7 g may be due to the decreased large S protein, direct effects of let-7 on cccDNA production, or indirect effects via let-7 g function on expression level changes of its target host genes. In addition, the fact that the sequence focused is in the overlapped sequences as templates for preS2 protein and polymerase makes the overall effects on cccDNA levels more complicated. The details of the effects on cccDNA by let-7 g expression or targeting the corresponding sequences need to be further determined.

Based on the results in this study, supplementation of let-7 g into infected hepatocytes may be beneficial to both the prevention of tumorigenesis and the inhibition of viral envelop protein production. Although LIN28B may not express a lot in non-inflammated or non-transformed hepatocytes, once it starts to transcribe, the post-transcriptional regulation must become crucial. The HBV large S transcript transgenic mice here did not show any spontaneous tumorigenesis without DEN treatment, suggesting that the targeted transcripts such as LIN28B mRNA need to be transcribed even at low levels to be targeted and modified post-transcriptionally. It may be important to determine which genes are indeed affected by the impaired host miRNA by the existence of HBV transcripts during the steps of chronic hepatitis and to determine the most appropriate timing for the supplementation of let-7 g into hepatocytes, to overcome the pathogenesis induced by the existence of HBV transcripts in hepatocytes.

In summary, we have shown that HBV preS2 transcript can be targeted by host cellular let-7 g, which may mutually anatagonize the intrinsic let-7 g function and HBV replication. Although the biological effects by the interaction between host and viral proteins on pathogenesis have been extensively studied, determining RNA-RNA interactions between the host and pathogens, and their biological roles, including viral intermediate transcripts, may shed new light on the pathogenesis of chronic pathogen infection.

## Methods

### Cell culture

The human HCC cell lines Huh7 and HepG2 cells were obtained from the American Type Culture Collection (ATCC, Rockville, MD, USA). HepG2.2.15. cells were obtained from Dr. Acs^20^. Cells were maintained in Dulbecco’s modified Eagle’s medium (DMEM) supplemented with 10% fetal bovine serum (FBS). Hep38.7 cells carrying the highest HBV replication levels, subcloned from HepAD38 cells, which are derived from a HepG2 cell line supporting tetracycline-inducible HBV replication, were kindly provided by Dr. Watashi^25^. The cells were maintained in Ham’s F-10/DMEM culture media supplemented with 10% doxycycline-free FBS with or without 1 μg/ml doxycycline to maintain the repression of HBV expression or to maintain HBV replication, respectively.

### Reagents

Doxycycline and doxycycline-free FBS were obtained from Clontech (Mountain View, CA, USA).

### Antibodies

Details are in the Supplementary Methods.

### Western blotting, transfection, and dual luciferase assays

Western blotting, transient transfection, and dual luciferase assays were performed as we described previously^46^.

### Plasmids

To construct the HBV large S mRNA-expressing plasmid, the large S region was amplified by PCR from genomic DNA of HepG2.2.15 cells, which harbor the genotype D HBV genome, was cloned into the pCDH vector (System Biosciences, Mountain View, CA, USA) by the In-Fusion cloning procedure (Clontech) at the NotI restriction site. To exclude the effects of protein products generated from the expression construct, we introduced point mutations to create stop codons just downstream of each translational start site of the large S, middle S, and small S proteins by mutagenesis using the Quik Change II Site-directed Mutagenesis Kit (Stratagene, Heidelberg, Germany), according to the manufacturer’s instructions. Additionally, to introduce mutations into the seed region putatively targeted by let-7, another mutagenesis was performed to introduce mutations (ACACUCCA to TCTCUCCA) into pCDH-large S–S, constructing pCDH-large S-SM.

The firefly luciferase-based reporter carrying let-7 g- and miR103-responsive elements in its 3′ untranslated region, to examine corresponding miRNA function (pGL4-let-7 g and pGL4-miR103), and the internal control renilla luciferase-based plasmids (pGL4-TK) have been described previously^47^. Let-7 g and miR103 precursor-expressing plasmids were constructed previously^48^,^49^. The pCDH control vector (System Biosciences) was used as a negative control. For transient assays, 2 × 10^5^ cells were seeded onto 6-well plates the day before transfection and 0.2 μg reporter plasmids (pGL4-let-7 g and pGL4-miR103) or empty vector (pGL4 vector) were transfected with 0.4 μg control vector (pCDH), pCDH-large S-S, or pCDH-large S-SM plasmids. When examining the effects of forced expression of miRNAs, 0.4 μg Let-7 g or miR103 precursor-expressing plasmids (pCDH-let-7 g or pCDH-miR103) were transfected simultaneously.

### Mouse experiments and construction of transgenic mice

Mouse experimental protocols were approved by the Ethics Committee for Animal Experimentation at the University of Tokyo (#13-P-54), and experiments were conducted in accordance with the Guidelines for the Care and Use of Laboratory Animals of the University of Tokyo.

To establish large S mRNA-expressing transgenic mice with and without mutations in the let-7 g-specific seed sequences, a DNA fragment of 2,535 bp, containing the CMV promoter region, the coding region of the large S mRNA, and a transcriptional terminator, was excised from the pcDNA3.1-Large S–S or pCDNA3.1-Large S-SM plasmids and subcloned into the EcoRI sites of pCDH-large S–S and pCDH-large S-SM by the In-Fusion method, as described above, by digestion with NruI and DraIII. Stable C57BL/6 embryonic stem (ES) cell lines were generated by electroporation of the linearized transgene, and the resulting cells were injected into blastocysts by the UNITECH Company (Chiba, Japan). Genotyping of DNA isolated from tail snips was performed by PCR. Primer sequences used for genotyping were 5′-AGG AGC AGT AAA CCC TGT TCC-3′ and 5′-CCT TGA TAG TCC AGA AGA ACC-3′.

### RNA isolation, reverse transcription, PCR analysis, *in vitro* translation, and Immunohistochemistry

Details are in the Supplementary Methods.

### *In silico* microRNA target prediction

Homologies between HBV sequences and human miRNAs were examined using the miRBase database^50^. The top 10 sequences with highest homologies were subsequently tested if they are miRNA targets in the RNA22 database based on the predicted thermodynamics^51^. Through these *in silico* selections, the highest probabilities were attributed to the HBV preS2 sequences and let-7 g.

### HBV sequence alignment

Representative HBV genotypes A, B, C, and D were selected by referring to a previous study^10^, and their sequences were extracted from the HBV sequence database, Hepatitis Virus Database (http://s2as02.genes.nig.ac.jp/index.html)^21^. The sequences were aligned in parallel using Genetyx 9 software. Information regarding the deletions and mutations in PreS regions was obtained from previous reports^23^,^24^,^43^,^44^.

### Ago2 immunoprecipitation and miRNA quantitation

Details are in the Supplementary Methods.

### DEN-induced experimental hepatocarcinogenesis

A single i.p. injection of 10 μg/g DEN in PBS was administered to the control, HBV-large S–S, and HBV large S-SM transgenic mice at 15 days of age. The mice were weaned at 3 weeks and kept in a temperature-controlled ventilated hood under a 12-h light-dark cycle, with free access to standard mouse chow and water. Transgenic and control mice (8/group) were sacrificed 16 weeks after DEN injection. The number and size of the grossly visible tumors were recorded. The liver sections were stained with H&E.

### Southern hybridization for HBV cccDNA

To extract cccDNA from the cells, a Hirt protein-free DNA extraction procedure^52^ was used. Briefly, after adding SDS to the cells, 1 M NaCl precipitated cellular chromatin and covalently bound proteins. Protein-fee DNA including cccDNA and deproteinized relaxed circular DNA were subsequently extracted from the supernatant by organic phenol.

To confirm equal DNA loading amounts, mitochondrial DNA was detected on the same membrane after stripping the probes. Primers AAC TAC GAA CGT ATT CAC AGC CG and GAA TTC TAT GAT GGA TCA GGT were used to generate the DIG-labelled probes against mitochondrial DNA^53^.

Detailed protocols for Southern blotting are in the Supplementary Methods.

### Statistical analysis

When the variances were equal, statistically significant differences between groups were identified using the Student’s *t*-test. When variances were unequal, Welch’s *t*-test was used instead. *P* values less than 0.05 were considered to indicate statistical significance.

## Additional Information

**How to cite this article**: Takata, A. *et al.* Mutual antagonism between hepatitis B viral mRNA and host microRNA let-7. *Sci. Rep.* **6**, 23237; doi: 10.1038/srep23237 (2016).

## Supplementary Material

Supplementary Information
