# Supplementary material for: Mutual antagonism between hepatitis B viral mRNA and host microRNA let-7
Source: Sci Rep. 2016 Mar 16;6:23237. doi: 10.1038/srep23237 (PMC4793232; doi:10.1038/srep23237)

Mutual antagonism between hepatitis B viral mRNA and host microRNA let-7

Akemi Takata, Motoyuki Otsuka, Motoko Ohno, Takahiro Kishikawa, Takeshi Yoshikawa, and Kazuhiko Koike

Supplementary Information Inventory

Supplementary Methods

Supplementary Figure Legends

Supplementary Figures (Supplementary Figures S1-S3)

- **S. Figure 1.** ***HBV preS2 mRNA can be targeted by let-7g***
- **S. Figure 2.** ***HBV preS2 mRNA inhibits let-7g function***
- **S. Figure 3.** ***Let-7g target protein expression in human HCC tissues***

Supplementary Methods

Antibodies

The antibodies anti-LIN28B (#11965), anti-c-Myc (#13987), and anti-HMGA2 (#8179) were purchased from Cell Signaling Technology (Danvers, MA, USA). Anti-HBV preS2 (ab30923) and anti-c-myc (ab32072) were purchased from Abcam (Cambridge, MA, USA). Anti-LIN28B (HPA061745) and anti-β-actin (#A5441) were purchased from Sigma (St. Louis, MO, USA). Anti-Ago2 antibody was purchased from Wako Chemicals (Osaka, Japan). Isotype IgG antibodies were purchased from R&D Systems (Minneapolis, MN, USA).

Viral transduction

When transducing two viral products, pCDH-hygro containing the hygromycin resistance gene was used for miRNA precursor expression. Lentiviral particles were produced using the pPACKH1 lentivector packaging plasmid mix, according to the manufacturers' recommendations (System Biosciences). Cells were transduced with lentiviruses using polybrene (EMD Millipore, Billerica, MA, USA), followed by selection with 6 and 2 µg/ml puromycin for Huh7 and Hep38.7 cells, respectively, and 400 µg/ml hygromycin for Huh7 cells.

RNA isolation, reverse transcription, and PCR analysis

Total RNA was isolated from cells using Trizol Reagent (Invitrogen). cDNA was synthesized from RNA using the SuperScript III First-Strand Synthesis System (Invitrogen). PCR amplification of regions of the large S transcripts was performed using LA-Taq polymerase (Takara, Shiga, Japan) and the primers ACC TCT ATG TATCCCTCCTG and GAC TCA AGA TGC TGT ACAGAC. PCR products were visualized after separation on

2.0% agarose/ethidium bromide gels. Primers used for amplifying the housekeeping gene GAPDH were TGC ACC ACC AAC TGC TTA G and GAT GCA GGG ATG ATG TTC.

In vitro transcription and translation

To confirm the effects of the newly introduced stop codons in the large S mRNA-expressing construct from pCDH-large S-S, in vitro transcription and translation were performed to confirm that the translation of the protein is inhibited, using the TNT Quick Coupled Transcription/Translation System (Promega). The large S region including the termination signal was amplified using primers, with the T7 promoter conjugated to the forward primer, and pCDH large S and pCDH large S-S as templates. The primers used were TAA TAC GAC TCA CTA TAG GG ATG GGG CAG AAT CTT TCC ACC and TTA AAT GTA TAC CCA AAG ACA. In vitro transcription and translation were performed according to the manufacturers' recommendations using Transcend Non-Radioactive Translation Detection Systems (Promega). After resolving the translated proteins by SDS-PAGE and transferring them to PVDF membranes, incorporated biotinylated lysines were reacted with HRP-conjugated streptavidin (GE Healthcare), followed by detection with ECL Plus Detection Reagents. As a positive control for these procedures, luciferase control DNA with the T7 promoter, included in the kit, was used in parallel according to the manufacturers' recommendations.

Ago2 immunoprecipitation

Ago2-related complexes were isolated using a human Ago2 microRNA isolation kit (Wako). Briefly, Ago2-related complexes were immunoprecipitated using anti-human Ago2 antibody according to the manufacturers' recommendations. Subsequently, miRNA was isolated from the precipitated complexes and quantitated.

miRNA quantitation

Real-time PCR for microRNAs, let-7g and miR103 was performed using the miRCURY LNA Universal RT microRNA PCR system (Exiqon, Vedbaek, Denmark) according to the manufacturer's instructions.

Immunohistochemistry

Tissue arrays containing HCC tissues infected with HBV or HCV (#LivT10-024) were purchased from US Biomax, Inc. (Rockville, MD, USA). The viral- and cancer-related clinical information of the patients is included with the samples in these arrays. Slides were baked at 65°C for 1 h and deparaffinized. Endogenous peroxidase activity was blocked by incubation in 3% hydrogen peroxide buffer for 30 min. Antigen retrieval was performed by incubating the slides at 89°C in 10 mM sodium citrate buffer (pH 6.0) for 30 min. To minimize non-specific background staining, slides were blocked in 5% normal goat serum (Dako, Glostrup, Denmark) for 10 min at room temperature. Tissues were incubated with primary antibodies overnight at 4°C. Slides were then incubated with horseradish-peroxidase-conjugated secondary antibodies (Nichirei Bioscience, Tokyo, Japan) for 1 h, followed by incubation in 3,3′-diaminobenzidine in buffered substrate (Nichirei Bioscience) for 5 min. The slides were dehydrated with ethanol and mounted with Clarion mounting medium (Biomeda, Foster City, CA, USA). LIN28 staining in HCC was evaluated and scored by two independent observers. Semi-quantitative scoring was performed based on the average signal intensity (0, no immunoreactivity; 1, weak intensity; 2, moderate intensity; and 3, strong intensity).

Southern hybridization for HBV cccDNA

For Southern blotting, 15 μg DNA were subjected to 1.2% agarose gel electrophoresis at 25 V overnight. Separated DNA on the gel was transferred to a Hybond-N+ membrane (GE Healthcare) in 20X SSC transfer buffer overnight. After transfer, the DNA was crosslinked by 120 mJ/cm^2 UV in a UV crosslinker (Stratalinker, Stratagene, La Jolla, CA, USA). To generate a HBV full length DNA probe for hybridization, the PCR DIG Probe Synthesis kit (Roche, Barsel, Switzerland) was used according to the manufacturers' recommendations. DIG-labelled dUTP was incorporated during the PCR amplification of HBV full length DNA, using HBV DNA sequences derived from HpeG2.2.15 cells as the template. Amplification of the correct product was confirmed by gel electrophoresis. The probe was hybridized to the membrane using DIG Easy Hyb (Roche) and detected using the DIG Wash and Block Buffer Set (Roche) according to the manufacturers' recommendations. Linearized HBV DNA, which was generated by PCR amplification of HepG2.2.15 genomic DNA, followed by gel-extraction, and heat-denatured Hirt-extracted small DNAs after incubating at 85°C for 5 min to denature DNAs, except cccDNA, were applied simultaneously as controls.

Supplementary Figure Legends

Supplementary Figure 1. HBV preS2 mRNA can be targeted by let-7g.

HBV sequences with homology to miRNAs were searched in the miRBase. The interactions of the top 10 candidate sequences with miRNAs were then confirmed using the RNA22 database based on the thermodynamic stabilities. Lower folding energy indicates a more stable interaction. Target nucleotide (nt) positions indicate the EcoRI site in HBV sequences as 1.

Supplementary Figure 2. HBV preS2 mRNA inhibits let-7g function.

a, HepG2 cells were transiently transfected with the indicated miRNA reporter constructs. When the corresponding miRNA precursor was overexpressed, the luciferase activity was significantly suppressed (shown as "Control"). Expression of Large S-S reversed such suppression by inhibiting let-7g function (left), but not in the case of miR103 (right). The Large S-SM construct did not show such effects. Data represent the means ± s.d. of three independent experiments, and the values from the cells without miRNA overexpression were set as 1. *, $p < 0.05$. **b,** Similar to (a) but without miRNA precursor overexpression. Large S-S inhibited endogenous let-7g function (left) but not miR103 function (right). Data represent the means ± s.d. of three independent experiments, and the values from the control were set as1. *, $p < 0.05$.

Supplementary Figure 3. Let-7g target protein expression in human HCC tissues.

a, Confirmation of Large S transcript expression in transgenic mouse livers. RNAs from liver were extracted, and RT-PCR was performed to confirm Large S transcript expression in Large S-S and Large S-SM transgenic mice. **b,** LIN28B protein expression statuses were determined by immunohistochemistry using human HCC and the surrounding tissues

derived from patients with HBV infection, along with their clinical information (HBeAg, HBsAg, and HBV-DNA load). Bar, 500 μm. **c,** Lin28B protein expression levels in human HCC tissues were determined with immunohistochemistry. The graph shows the difference in Lin28B expression levels among HBeAg-, HBsAg-, and HBV-DNA- positive or negative patients.

Supplementary Figure S1

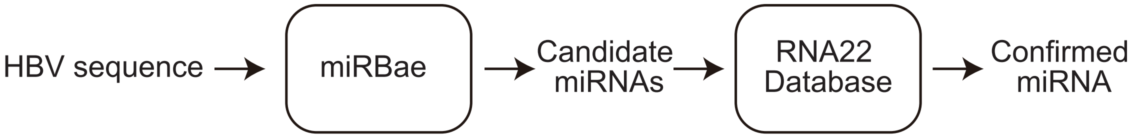

miRBae		RNA22 Database	
microRNA	Seed sequence homology	Folding energy	Target nt position
let-7g	87.5%	-24.2 Kcal/mol	88
miR-16	75%	-	
miR-30c-2*	75%	-24.1 Kcal/mol	3149
miR-573	75%	-	
miR-557	75%	-17.8 Kcal/mol	201
miR-199b-5p	75%	-16.5 Kcal/mol	1712
miR-515-5p	75%	-	
miR-514	75%	-	
miR-450b-5p	62.5%	-	
miR-214	62.5%	-12.7 Kcal/mol	400

Supplementary Figure S2

a

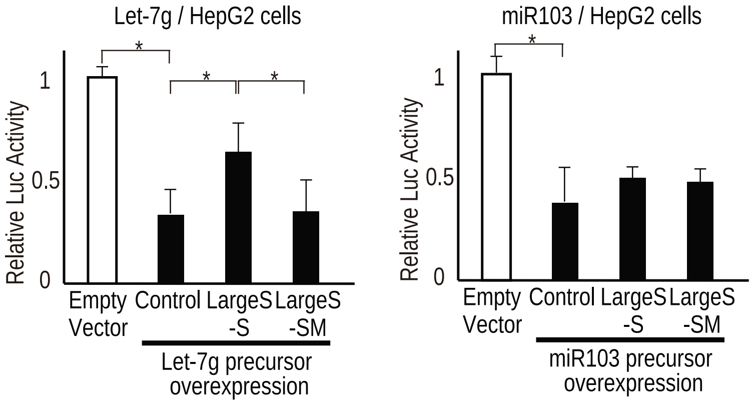

b

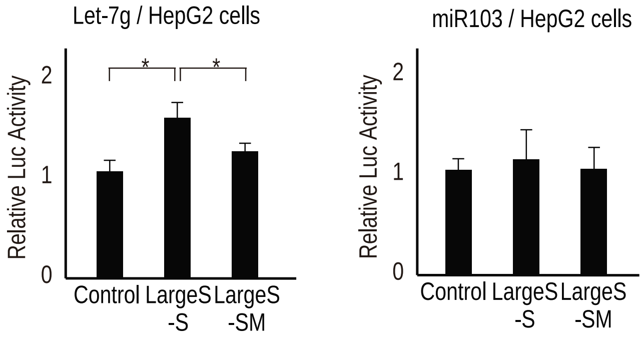

Supplementary Figure S3

a

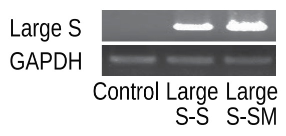

b

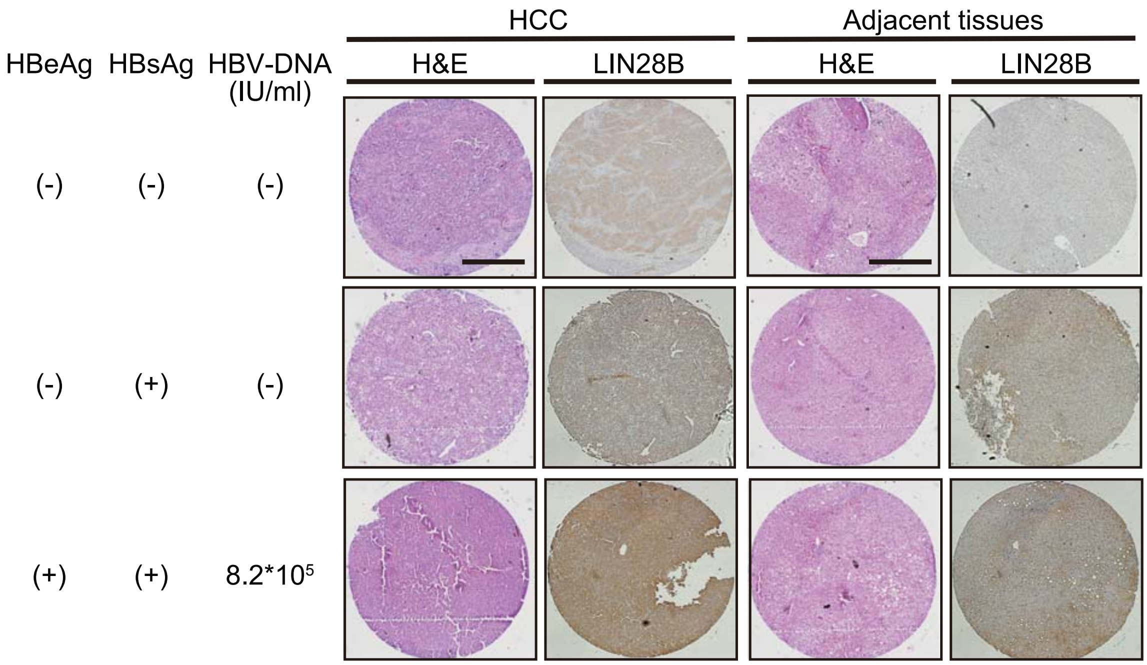

c

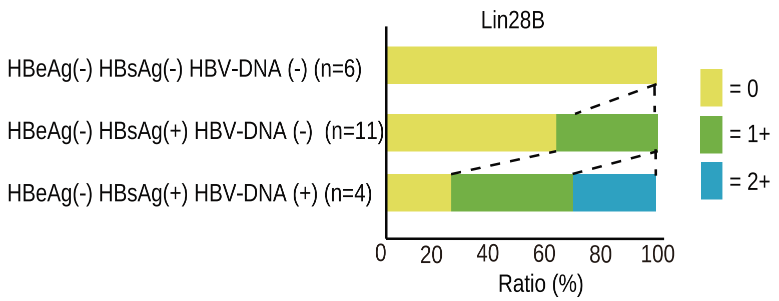

Supplement: Supplementary Information [file srep23237-s1.pdf]
